# Horizontal spin of ratchet motor by vertical agitation

**DOI:** 10.1038/s41598-021-91319-8

**Published:** 2021-06-07

**Authors:** Toshinobu Takahashi, Erika Okita, Daigo Yamamoto, Yasunao Okamoto, Akihisa Shioi

**Affiliations:** 1 Department of Chemical Engineering & Materials Science, Doshisha University, 1-3 Tatara Miyakodani, Kyotanabe, Kyoto 610-0321 Japan; 2 Department of Chemical Engineering, Osaka Prefecture University, 1-1 Gakuen-cho, Naka-ku, Sakai, Osaka 599-8531 Japan

**Keywords:** Applied physics, Chemical physics, Statistical physics, thermodynamics and nonlinear dynamics

## Abstract

The horizontal spin of a ratchet motor by vertical vibration is reported. A macroscopic ratchet gear is placed on a granular bed, where nearly half of the gear is penetrated in the bed. The gear and granular bed are mechanically vibrated. The gear shows a random motion or one-way spin that depend on the diameter of the granules, vibration frequency, and degree of vertical motion allowed for the gear. Even when one-way spin is observed, the spin direction depends on the abovementioned factors. Although the dependency is complicated, it is deterministic because the motion or flows of granular matter determines it. The characteristics observed in the experiments are explained by a simple model that accounts for the statistical variance in the motion of the granular matter. Extraction of systematic motion from small and non-useful motions such as mechanical agitation will be developed into energy harvest technology and may facilitate the science of a spontaneously moving system in a uniform potential field.

## Introduction

The second law of thermodynamics states that one cannot obtain mechanical work from uniform thermal motion. It is believed that the thermal Brownian motion comprises numerous straight movements with extremely short distances^1,2^. In fact, the mean square displacement (MSD) of a colloid particle with Brownian motion exhibits $\mathrm{MSD}\propto {t}^{2}$, when the time (*t*) is less than ${10}^{-7}-{10}^{-8}$ s^1,2^. This is characteristic of ballistic motion. Meanwhile, random motions caused by nonequilibrium processes such as chemical reactions and mechanical agitations can be transformed into systematic motions^3–7^. In particular, the former is a key phenomenon for biological motions^8,9^, and both may be utilized for energy harvest technology. As biological motions are considered for harvesting mechanical works using thermal agitation under uniform temperature^8,9^, biological systems may be regarded as the most efficient energy harvest system.

One can obtain a systematic motion such as a one-way translation and spin from self-propelled colloids^10^, living microorganisms^11^ and mechanical agitations^4,7^. The uniform thermal motion under thermal equilibrium is meaningless from the technological perspective, whereas a similar motion under nonequilibrium state is useful for generating a systematic motion (mechanical work). Even when the random motion is caused by nonequilibrium processes, an optimal condition must be satisfied for transformation into systematic motion^12^. Outside the optimal conditions, one only regards them as random and meaningless motions. The optimal conditions have been investigated in many systems, particularly in self-propelled colloids driven by chemical processes^10,12^. In most cases, the optimal conditions are restricted in a narrow range; hence, the resulting motion often exhibits a stimuli responsive nature, similar to biological motions.

Transformation from weak mechanical agitation into systematic motion is easier to discuss compared with chemically driven systems, as neither chemical reactions, ad/desorption, nor diffusion are involved. The physics of energy transformation with the periodic potential changes in space and time have been studied deeply^13–17^ and inspire the design of artificial molecular motor^18^. However, studies regarding mechanical agitation systems with macroscopic scale are fewer^19–21^ than those regarding chemically driven systems. The dynamical behavior of granular matter on a vibrating disk have been investigated for a significant amount of time. Numerous nontrivial phenomena have been reported, such as segregation of larger particles and convections, based on boundary conditions^22–26^. These results suggest that granular matter can transform vertical agitation into horizontal systematic motion. Studies regarding the optimal condition and mechanism for this energy transformation will be useful for elucidating how one can obtain a systematic motion from its orthogonal agitations. This may be fundamental for utilizing mechanical vibrations in energy harvest technology.

In this study, the spinning motion of a gear in granular matter was investigated. The granules and gear were placed on a vibrating disk. The gear exhibited one-way spin and almost random motion depending on the experimental conditions, such as the gear shape, size of granules, and vertical moving range allowed for the gear. The motion type and spin direction depended significantly on the experimental conditions. Although the behavior appeared to be stochastic, it was deterministic and well reproducible. A mathematical model for the gear motion reveals the stochasticity of this deterministic process.

## Results

### Spinning gear

**Figure 1:**
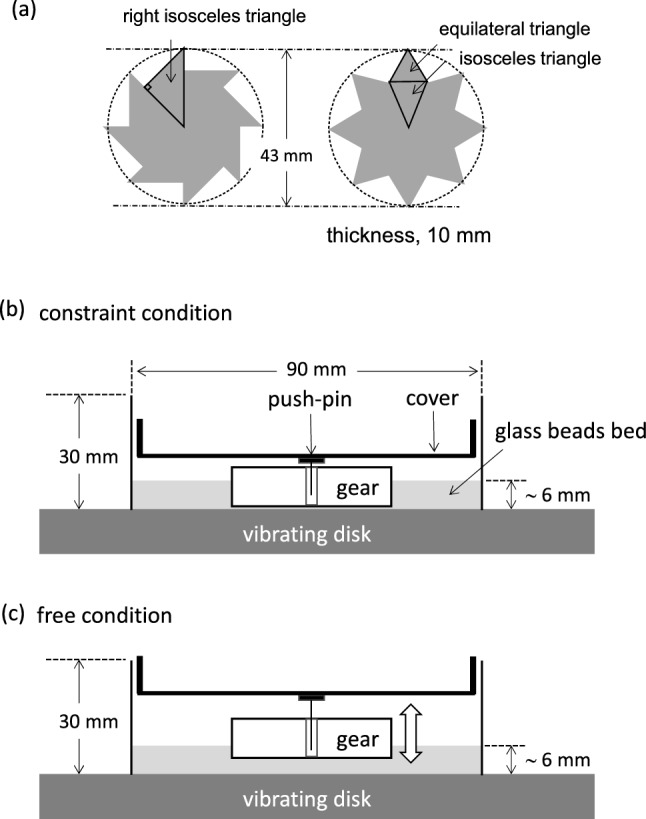
Schematic representation of experimental setup. (**a)** Asymmetric and symmetric gears; (**b**,**c**) setup for constraint (**b**) and free (**c**) conditions.

Figure 1a shows the gears used for this experiment. The gear shape is based on the study of Leonardo et al.^11^. Two types of gears were used, i.e., symmetric and asymmetric ones. The geometry and size are shown in Fig. 1. The gear was placed in an acrylic Petri dish filled with 50 g glass beads. The gear center was fixed using a push-pin fixed to a vertically vibrated cover.

**Figure 2:**
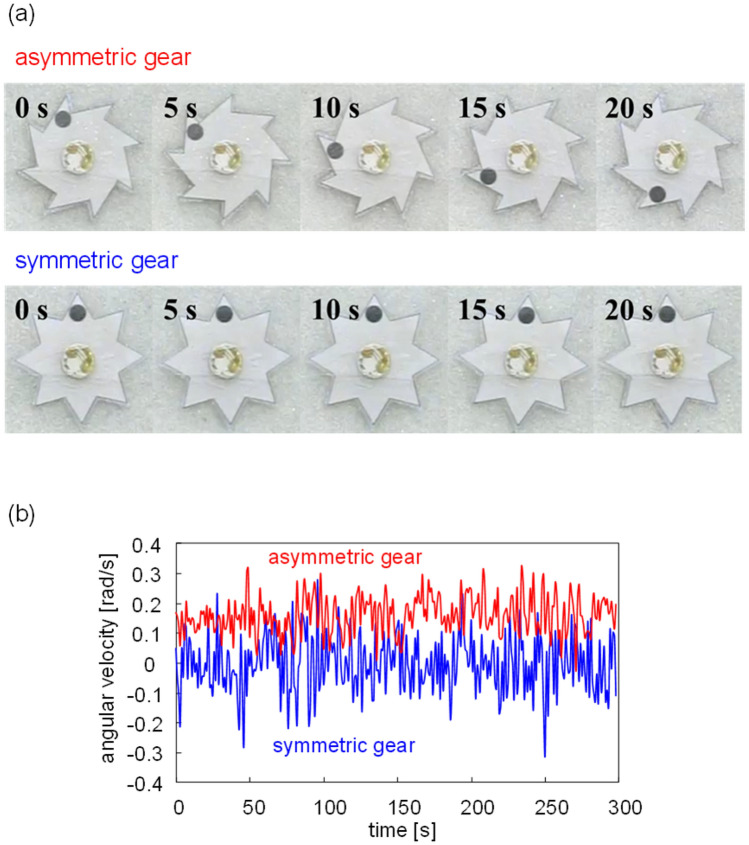
Motion of gear. (**a**) Snapshots of asymmetric and symmetric gears; (**b**) time course of angular velocity with bead diameter of 0.6 mm under 30 Hz.

First, the experiment was performed such that the vertical motion of the gear was significantly restricted, as shown in Fig. 1b. This experiment was regarded as the constraint condition. The other experiment, in which the gear can vibrate freely, was regarded as the free condition (Fig. 1c). Figure 2a shows snapshots of the moving gears with symmetric and asymmetric shapes in the constraint condition. (Supplementary Movie 1, for symmetric and asymmetric gears.) For all experiments in the present study, the symmetric gear exhibited random motions, where the spinning direction changed many times occasionally, as shown in Fig. 2b, where the angular velocity of the symmetric gear fluctuated around zero. Meanwhile, the asymmetric gear demonstrated a one-way spin. The spinning direction of the asymmetric gear shown in Fig. 2a,b is anti-clockwise (a positive angular velocity implies an anti-clockwise spin). However, the direction depends on the experimental conditions, such as the constraint or free conditions and the diameter of the glass beads. Hereafter, the results of the asymmetric gear are discussed because the one-way spin is focused more in this study.

**Figure 3:**
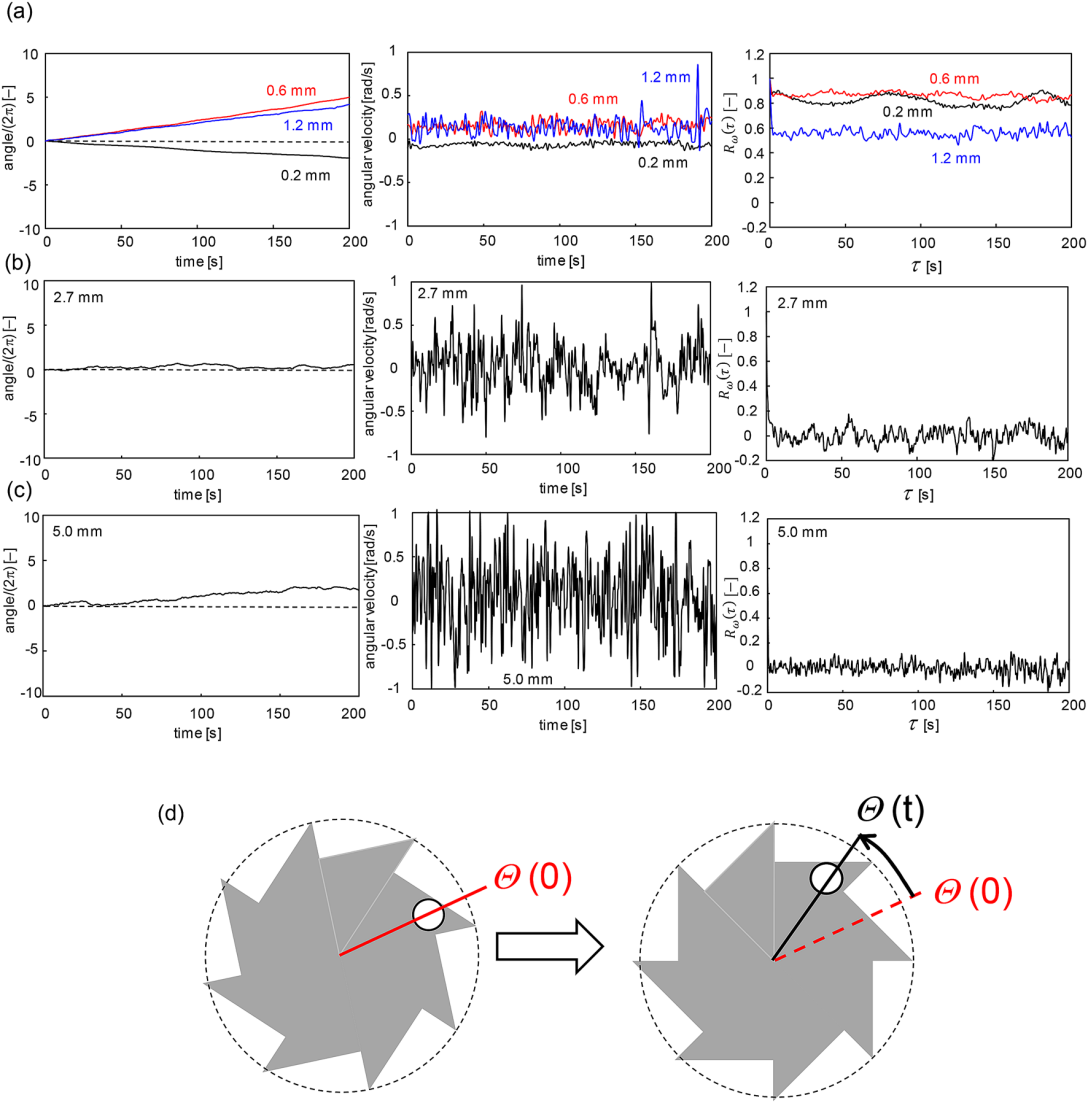
Characteristics of gear motion with constraint condition: azimuth change, angular velocity, and autocorrelation function for bead diameters of (**a**) 0.6–1.2 mm, (**b**) 2.7 mm, and (**c**) 5.0 mm. (**d**) Definition of azimuth change *Θ*.

Figure 3a shows the time course of the azimuth change of a point positioned at the tooth tip of an asymmetric gear (black circle shown in Fig. 2a). The azimuth change defined as *Θ* in Fig. 3d increases by 2π with its one round spin, and the ordinate of Fig. 3a is expressed by the azimuth change divided by 2π. Here, the result with the constraint condition is shown. The frequency of the vibrator is 30 Hz. The angular velocity and its autocorrelation function (${R}_{\omega }\left(\tau \right)$) is shown as well. The autocorrelation is calculated by

$${R}_{\omega }\left(\tau \right)=\frac{\sum_{i=1}^{N}\omega \left({t}_{i}\right)\omega \left({t}_{i}+\tau \right)}{\sum_{i=1}^{N}\omega \left({t}_{i}\right)\omega \left({t}_{i}\right)}$$

Here, *ω*(*t*) is the angular velocity at time *t*. The start time ${t}_{i}$ was selected from one experimental result. The number of the start time (*N*) was 300. When the bead diameter was less than 1.2 mm, the angle increased or decreased monotonically, and the sign (+ / −) of the angular velocity remained almost unchanged. These results indicate the one-way spin. The spin direction for the smallest bead (0.2 mm) was clockwise, opposite to those of the 0.6 and 1.2 mm beads. For the larger beads (2.7 and 5.0 mm in diameter), the angular velocity fluctuated around zero, and the azimuth did not exhibit a monotonous change. For these larger beads, the spin direction changed often; hence, it was not a one-way spin. The autocorrelation function of the angular velocity clearly demonstrated the effect of the bead diameter. For bead diameters less than 1.2 mm, ${R}_{\omega }\left(\tau \right)$ did not decay over 200 s. By contrast, for the larger beads, ${R}_{\omega }\left(\tau \right)$ decays within a very short time. The dependency of spin direction on the bead diameter was well reproducible.

**Figure 4:**
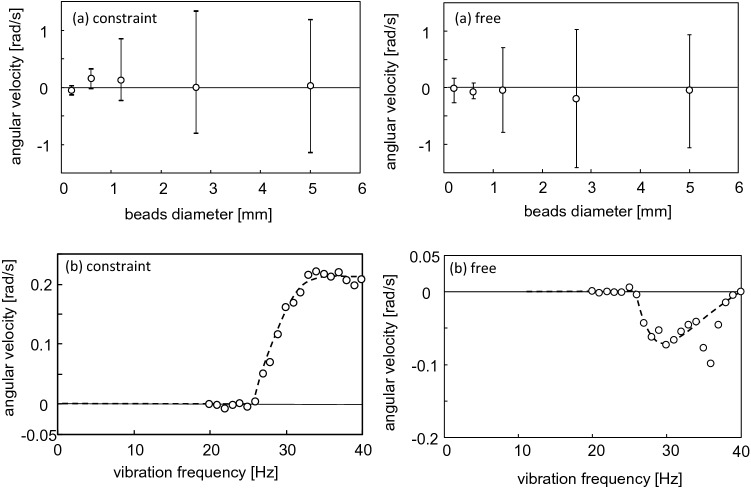
Angular velocities of constraint and free conditions shown as a function of (**a**) bead diameter (for frequency = 30 Hz) and as a function of (**b**) vibration frequency (for bead diameter = 0.6 mm).

Figure 4a (constraint) shows the dependency of the angular velocities at 30 Hz (its average, the maximum, and the minimum for 300 s) of the constraint condition on the bead diameter. The average angular velocity was the maximum for a diameter of approximately 0.6 mm. The range of the angular velocity expanded monotonically as the bead diameter increased. This indicates that the gear motion randomized with an increase in the bead diameter, and that the angular velocity was the maximum at a diameter of approximately 0.6 mm. Figure 4b (constraint) shows the average angular velocity with 0.6 mm beads as a function of vibration frequency. It increased steeply beyond the threshold (approximately 25 Hz) to reach a plateau, at approximately 0.2 rad/s.

The same experiments were performed with an asymmetric gear for the free condition. The results corresponding to Fig. 3a–c are provided in the supplementary information (Fig. S1), and the results corresponding to Fig. 4a,b (constraint) are shown in Fig. 4a,b (free), respectively. For all bead diameters, the average angular velocity was negative, indicating a clockwise spin. As the bead diameter increased, the absolute value of the average angular velocity increased to reach the maximum at approximately 2.7 mm. Until this maximum, the range of angular velocity increased. For the dependency of the vibration frequency, a threshold value for the spin was observed at approximately 25 Hz, which was the same value as that for the constraint condition. At the higher frequency, the absolute value of angular velocity increased, as observed for the constraint condition. However, the average angular velocity (absolute value) at a frequency higher than 30 Hz began to scatter and appeared to decrease. For the free condition, the vertical vibration of the gear was allowed. When the vibration frequency was excessively high, this vertical motion became outstanding. Subsequently, the gear ejected from the granular bed. In this case, the spinning motion of the gear was unstable, and its angular velocity (absolute value) decreased. This might have caused the angular velocity to be primarily scattered and demonstrate a decreasing trend for the high-frequency region.

The spin direction changed based on the bead diameter and the constraint or free conditions; the small change in the experimental conditions changed the motion significantly. However, the spinning direction and the angular velocity were highly reproducible. Therefore, they were deterministic in nature.

### Motion of glass beads

**Figure 5:**
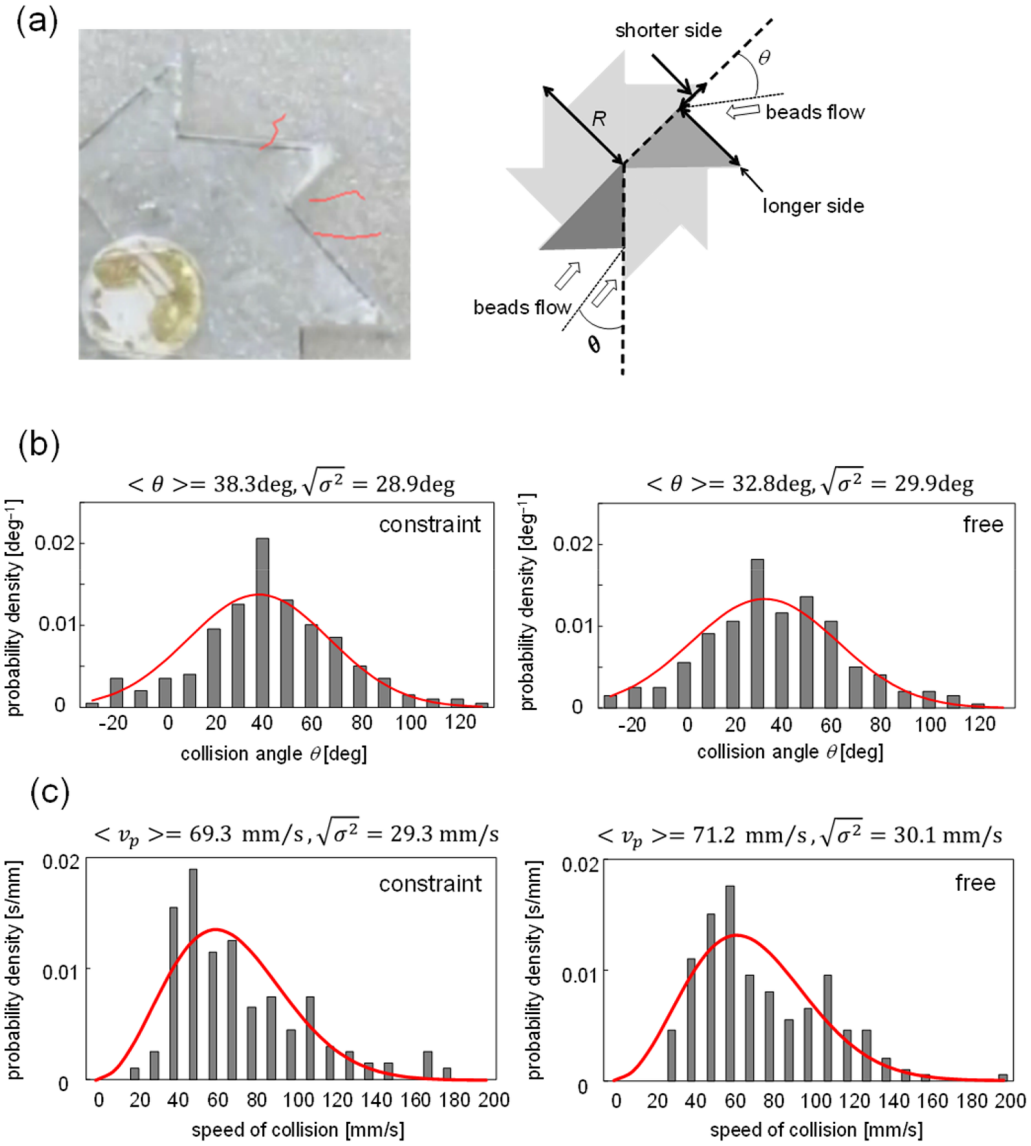
Characteristics of bead motion. (**a**) Examples of trajectory of beads with 0.6 mm diameter under 30 Hz, and definition of collision angle *θ*. (**b**) Distribution of collision angle. (**c**) Distribution of collision speed. Solid curve shows approximation by (**b**) normal and (**c**) Maxwell–Boltzmann distributions. Average and dispersion are shown at the top.

The glass beads were agitated by the vibration to exhibit the convective motion. Figure 5a exemplifies the trajectories of three colored beads on the surface of the granular bed. The bead diameter was 0.6 mm, and the frequency was 30 Hz. (Supplementary Movie 2) The beads collided with the gear wall and penetrated into the granular bed: The convective motion occurred within the dent of the gear teeth, i.e., the bead motion was generated by the effect of the moving gear wall. Moreover, the beads in the convective motion collided with the gear wall at various velocities (the incident angles and speeds). Figure 5b,c show the distribution of the incident angle and the speed of the collided beads, respectively, where the angle *θ* is defined as shown in Fig. 5a. Both distributions were calculated from the experiments using diameters of 0.6 and 2.7 mm. In this study, we used the result of 50 collisions for the 0.6 and 2.7 mm diameters. These two diameters were selected as typical cases for the smaller and larger beads, respectively. Figure 5b,c show the summation of the results for both diameters. Both the distributions of the angle and speed changed slightly based on the conditions (constraint or free). For the free condition, the collision angle was slightly smaller, and the speed was slightly higher.

### Model

Consider an asymmetrical gear that exhibits a one-way spin (see the right isosceles triangles highlighted in Fig. 5a). These two triangles are merely examples for all the triangles. Consider the beads colliding with the gear wall by an angle *θ*, as shown in Fig. 5a. When the velocity is *v*_p_, the force imposed on the unit length of the colliding surface (pressure) (*p*) is expressed as

1 $$p = \frac{{m_{p} v_{p} }}{{d_{p} \Delta t}}$$

Here, ${m}_{p},$ *d*_p_ and *Δt* represent the mass of one bead, the bead diameter and impulse time, respectively. The components of the the longer and shorter sides shown in Figs. 5a and S2, respectively, are given by ${\text{p}}{\mathrm{cos}}\theta\space \tt \, and \,\space {\text{p}}{\mathrm{sin}}\theta .$ The moment of force (torque) *T* to spin the gear generated by the pressure is expressed as

2 $$T = 8pR^{2} \left( {\frac{1}{4}\sin \theta - \frac{\sqrt 2 - 1}{2}\cos \theta } \right)$$

The derivation is shown in Fig. S2 in the supplementary information. The pressure *p* is calculated using Eq. (1).

In the present experiment, the resistance for the gear spin was strong because part of the gear was in the granular bed. Hence, the inertia term in the equation of spin motion may be neglected. Subsequently, the equation of motion is expressed as shown in Eq. (3).

3 $$T = k_{i} \frac{d\Theta }{{dt}}$$

Here, *k*_i_ is the coefficient of the resistance against the spinning motion, and *Θ* is the azimuth change shown in Fig. 3d. The resistance coefficient is expressed as shown in Eq. (4).

4 $$\begin{aligned} & k_{c} = 8\left( {\frac{4 - \sqrt 2 }{{12}}} \right)\frac{{m_{p} R^{3} }}{{d_{p} \Delta t}}\;\;\;{\text{for}}\;{\text{cloclwise}}\;{\text{spin }}, \\ & k_{ac} = 8\left\{ {\frac{1}{4} + \frac{\sqrt 2 }{8}\ln \left( {1 + \sqrt 2 } \right)} \right\}\frac{{m_{p} R^{3} }}{{d_{p} \Delta t}}\;\;\;{\text{for}}\;{\text{anticlockwise }}\;{\text{spin}} \\ \end{aligned}$$

See Fig. S3 in the supporting information. These equations were derived from the torque required for the gear spinning in a motionless bed: We assumed that the collision of the gear face toward the motionless bed dominated the resistance.

Subsequently, Eq. (3) was numerically integrated using Eqs. (1), (2), and (4). In this calculation, *m*_p_, *d*_p_, and *Δt* were cancelled because they were included with the same order on both the right and left sides of the equation. Hence, the bead diameter did not affect the calculation result. However, it affected the calculation through the distribution of ${v}_{p}$ and $\theta$ shown in Fig. 5b,c. A large number of beads collided with the gear wall almost simultaneously. Therefore, the ${v}_{p}$ and $\theta$ used for Eqs. (1) and (2) should be the averages for the numerous collisions of beads. Consider the normal distribution with average *μ* and dispersion *σ*^2^. The normal distribution may be used for the collision angles. When a sample with size *n* (number of beads) are extracted from this distribution (population distribution), the average of this sample follows the distribution with average *μ* and dispersion *σ*^2^/*n*. Consider the Maxwell–Boltzmann distribution with the dispersion *σ*^2^. (The *μ* and *σ*^2^ are related to each other.) The Maxwell–Boltzmann distribution may be used for the speeds. When a sample with size *n* (number of beads) is extracted from this distribution (population distribution), the dispersion of the average becomes *σ*^2^/*n*. This indicates that the ${v}_{p}$ and $\theta$ used for Eqs. (1) and (2) follow the distribution with the dispersion *σ*^2^/*n*, where *n* is the number of beads colliding simultaneously with the gear wall (within *Δt*). This number of beads must be proportional to *d*_p_^-2^, because the area of gear wall colliding with the beads is the same irrespective of the bead diameter. Therefore, the bead diameter affects the calculation through its effect on the dispersion.

For the integration of Eq. (3) with Eqs. (1), (2), and (4), the population distributions for *θ* and *v*_p_ must be known. The distributions that can fit the experimental distribution is shown in Fig. 5b,c, where the average and dispersion values are shown. The normal and Maxwell–Boltzmann distributions are used for *θ* and *v*_p_, respectively. The distributions of the collision angle and speeds were dependent on the bead diameter, as shown in Fig. S4. For both the distributions of the angle and speed, the dispersions for the 2.7 mm diameter were larger than that for the 0.6 mm diameter. This was likely because only the particles on the surface of granular bed can be tracked. The bead motions were averaged by the numerous interparticle collisions in the granular bed. However, this averaging effect was weaker on the surface of the granular bed, where the bead trajectory can be tracked. The larger bead possessed larger inertia. Therefore, the effect of interparticle collision was not reflected in the larger particles on the surface of the granular bed. This might have resulted in the larger dispersions for the results of the 2.7 mm diameter, as shown in Fig. S4. To obtain the population distributions of the angle and speed, the measurement of the bead motion inside the granular bed is required. However, it is difficult to observe the bead motion in the bed. Therefore, as an approximation, the results of both diameters were summed to evaluate the population distribution.

The dispersions of the collision angle and speed were evaluated from *σ*^2^/*n*, where the *σ*^2^’s for the angle and speed were those shown in Fig. 5b,c, respectively. Two parameters, $<\theta >$ and *n*, were selected as adjustable parameters, because these two parameters affected the calculation result significantly. Hence, the distributions for the angle and speed used for the calculation of Eqs. (1) and (2) were determined, and the numerical integration of Eq. (3) can be performed without more adjustable parameters (*R* is the gear radius; 21.5 mm). At each step of the numerical integration, *θ* and *v*_p_ were assigned random numbers following the corresponding distributions. In the experiments, the azimuth was measured every 1 s. Therefore, for the numerical integration of Eq. (3), each step was regarded as 1 s for comparison with the experimental results.

**Figure 6:**
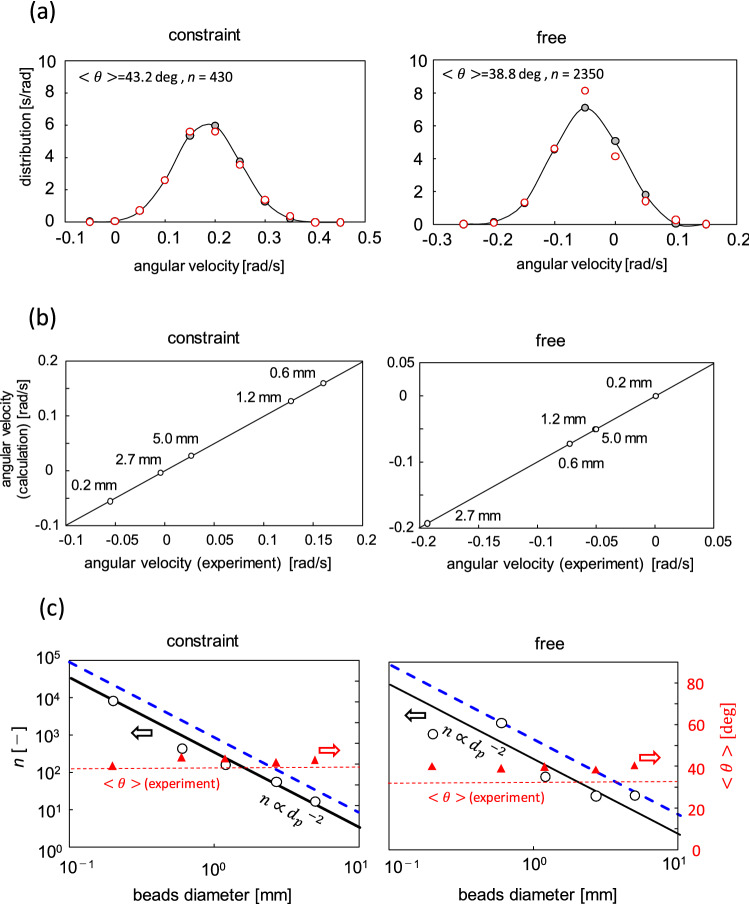
Calculation results. (**a**) Distribution of angular velocity for constraint and free conditions. Calculation result shown by filled circle with fitted curve. Experimental results with 0.6 mm diameter shown by open circle. (**b**) Average angular velocity obtained by calculation and experiment. In right panel, the keys of 1.2 and 5.0 mm are overlapped. (**c**) Values of adjustable parameters shown by triangle (< *θ* >) and circle (*n*). Dotted horizontal line shows experimental value of average collision angle; dashed line shows maximum value of *n* calculated using bead diameter and gear wall area.

The time variation of the azimuth (*Θ* in Fig. 3d) was calculated by the integration of Eq. (3), where the torque *T* was calculated using the distributions of *θ* and *v*_p_ and Eqs. (1), (2) and (4). The distribution of angular velocity was evaluated from this time course of the angle. Figure 6a shows the results calculated for reproducing the experimental result with 0.6 mm diameter. Both the constraint and free conditions were calculated. The same results for the other beads diameters are shown in Fig. S5 both for constraint and free conditions. The distributions experimentally observed for the angular velocity were well reproduced by the present model. The average angular velocities of the experiment and calculation are compared in Fig. 6b. The present calculation can explain the angular velocity as well as its average and distribution, by adjustable parameters $<\theta >$ and *n*.

Figure 6c shows the values of $<\theta >$ and *n* that can reproduce the experimental angular velocity. The $<\theta >$ for each diameter agreed approximately with that observed from the experiment, particularly in the constraint condition. (Here, < *θ* > _(experiment)_ is the average of the results with 0.6 and 2.7 mm diameters. The differences between the both cases is approximately 3.8 deg (constraint) and 2.1 deg (free).) For the free condition, the agreement was worse. However, the difference was 4° at the maximum, which was small. The value of *n* is proportional to *d*_p_^-2^. The maximum value of *n* is considered to be equal to the area of the gear wall divided by the cross-sectional area of the bead, where the gear wall area is the area of the gear wall that penetrated in the granular bed. This maximum value of the bead number was calculated with 6 mm depth, as shown in Fig. 1b by the dashed (blue) lines. Figure 6c for the constraint condition shows that the *n*-value was approximately one-fifth the maximum value. Because all the beads adjacent to the gear wall did not collide with the wall, the result is reasonable. In the free condition, the gear can move vertically. Therefore, the depth of the wall that penetrated into the granular bed fluctuated. The *n* value that can reproduce the experimental results might fluctuate for this reason. The larger fluctuation observed in the result of the free condition shown in Fig. 6c likely reflects the vertical vibration of the gear.

The collision angle that resulted in *T* = 0 was 39.6°, which was similar to the < *θ* > , which can reproduce the experimental results (Fig. 6c). (This angle is calculated by the driving torque shown in Fig. S2, where the resistance torque shown in Fig. S3 is not considered, because the gear does not spin under the zero torque.) The convective motion of the beads was formed such that the average collision angle was similar to the angle of the zero torque. For the symmetric gear, it did not spin in one direction, indicating that the bead collided with the gear wall by the collision angle with the zero torque. In the present study, when the gears were placed on the vibrating granular bed, the convective motion of the beads developed, causing them to collide with the angle of the zero torque. This phenomenon must be understood such that the reversed spin direction of the 0.2 mm diameter in the constraint condition can be clarified. Although the average collision angle < *θ* > was similar to the angle of the zero torque, the angle depended slightly on the bead diameter. For the bead with 0.2 mm diameter in the constraint condition, the average collision angle decreased slightly, as shown in Fig. 6c. However, when the average collision angle was less than 39.6° by this decrease, the spin direction was reversed.

It is essentially important for explaining the gear spin by the present model that the beads collide with the gear wall nearly the angle with zero torque. The reason why such beads convection is developed is unclear at present and an interesting research topic: The whole system of the gear and the beads may prefer the spin-less state (zero torque).

The distribution of the collision speed shown in Fig. 5 may be fitted by the normal distribution. The fitting is worse as shown in Fig. S6. The difference in the dispersions obtained by the normal and by Maxwell–Boltzmann distributions is within 5–15%. (See the dispersions shown in Figs. 5 and S6.) The calculation results corresponding to Fig. 6 with the normal distribution was identical to Fig. 6. (The agreement was perfect, at least, within three active digits.) The type of distribution function does not affect the conclusion of this study.

The population distribution was estimated by the summation of the results of 0.6 and 2.7 mm diameters. However, the conclusion above that the value of *n* is proportional to *d*_p_^-2^ is not affected by this approximation provided that the population distribution does not depend on the bead diameters.

## Discussion

When the gear used in the present study was placed on a vibrating disk without the granular bed, the gear moved randomly in the horizontal and vertical directions. However, the asymmetric gear can exhibit the one-way spin in the vibrating granular bed. For the one-way spin, the frequency must be beyond 25 Hz. The period for which the gear maintains the one-way spin depends significantly on the bead diameter. The smaller bead measuring less than 1.2 mm in diameter exhibited the one-way spin. However, the spin direction was reversed when the gear can move vertically. Moreover, in the constraint condition, the spin direction with the 0.2 mm bead diameter was opposite to those with the 0.6 and 1.2 mm diameters. The bead moved on the vibrating disk, and the convective motion was regulated by the gear shape. When the bead collided with the gear wall, the average collision angle was similar to the angle where the torque spinning the gear became zero. However, the average collision angle depended slightly on the bead diameter and the degree of the vertical motion of the gear. When this slight change in the collision angle occurred and crossed the angle of the zero torque, the spinning direction is reversed.

For the larger bead diameter, the spin direction changed frequently. Consequently, the motion appeared random. The beads with the various collision angles and speeds collided with the gear wall. For the statistical dispersion, the average collision angle formed by the simultaneous collision changed with time, and the average collision angle scattered around the true average generated by the collisions of the beads. In other words, the average collision angle fluctuated around the true average. The number of beads that simultaneously collided with the gear wall was smaller for the larger bead diameter. The smaller number of beads resulted in the larger fluctuation of the average collision angle and speed. Therefore, the angular velocity of the gear fluctuated significantly for the beads with the larger diameters. As the true average of the collision angle was similar to the angle with zero torque, the gear spin direction changed frequently for the larger diameter beads.

Small vibrations are ubiquitous in our lives, and they are primarily converted to thermal energy. Utilizing the granular bed appropriately, one may be able to obtain a systematic motion from small vibrations. However, its control requires the knowledge regarding the dynamics of granules in the vibrating environment. Under non-equilibrium conditions, any objects, from molecules to macroscopic objects, can move spontaneously. However, the motion is random unless a systematic gradient appears in the potential field. The transformation of a random motion to a systematic one is key for obtaining the regulated motion in an apparently uniform field. This applies to biological motions as well. The present study may be useful for future studies pertaining to such energy transformations.

## Methods

Figure 1a shows the gears used for this experiment. The gear shape used was based on that by Leonardo et al.^11^. Two types of gears were used, i.e., symmetric and asymmetric ones. The geometry and size are shown in Fig. 1a. Both were fabricated using 10-mm-thick silicon rubber. The gear was placed in an acrylic Petri dish with 90 mm diameter and 30 mm depth, as shown in Fig. 1b,c. Glass beads weighing 50 g were placed in the Petri dish. They were fully dried in a dryer before the experiments. The diameter of the beads were 0.2, 0.6, 1.2, 2.7, and 5.0 mm. The gear was placed on the bead bed at the beginning. An acrylic cover was placed on the Petri dish. This cover was fixed to the Petri dish to inhibit relative motions. A push-pin was glued at the center of the cover. The tip of the pin was inserted into the hole at the center of the gear to avoid its horizontal motion. The diameter of the center hole was 3 mm. Although this diameter allowed a slight horizontal motion of the gear, this allowance was required for the spinning motion of the gear. The Petri dish in which the beads and the gear were set was placed on a disk. Vertical vibration was applied to this disk. The vibrator (513-B, EMIC Co.) comprised a function generator (DF1906, NF Co.) to generate a sinusoidal wave, and an accelerometer with a charge amplifier (505-CBP, EMIC Co.). The amplitude was controlled to 1.8 mm using a power amplifier (371-A/G, EMIC Co.).

The vibrating disk where the Petri dish was fixed onto was vertically agitated at a predetermined frequency and the amplitude. Two types of setups were used. One involved the constraint condition (Fig. 1b), where the vertical motion of the gear was restricted significantly. Almost no gap was observed between the gear top and the cover. The other involved the free condition (Fig. 1c), where the gap was sufficiently large to allow the unrestricted vertical motion of the gear. The gap was approximately 3 mm, which was sufficient for the unrestricted vertical motion of the gear.

The spinning motion of the gear was monitored using a digital camera (CASIO EX-100F), and the position of a fixed point at the tip of the gear was traced using Move—Tr/2D (Library Co.) software. Its azimuth was measured and then used to calculate the angular velocity. For tracking the beads motion, several beads were colored and tracked by the software.

## Supplementary Information

Supplementary Information 1.

Supplementary Movie 1.

Supplementary Movie 2.
