# Supplementary material for: Horizontal spin of ratchet motor by vertical agitation
Source: Sci Rep. 2021 Jun 7;11:11983. doi: 10.1038/s41598-021-91319-8 (PMC8185098; doi:10.1038/s41598-021-91319-8)
Supplement: Supplementary file 1 — Supplementary Information 1. [file 41598_2021_91319_MOESM1_ESM.pdf]

Horizontal Spin of Ratchet Motor by Vertical Agitation

Toshinobu Takahashi[1], Erika Okita[2], Daigo Yamamoto[1], Yasunao Okamoto[1], and Akihisa Shioi[1*]

1 Department of Chemical Engineering & Materials Science, Doshisha University
1-3 Tatara Miyakodani, Kyotanabe, Kyoto 610-0321 Japan

2 Department of Chemical Engineering, Osaka Prefecture University
1-1 Gakuen-cho, Naka-ku, Sakai, Osaka 599-8531 Japan

(*)e-mail: ashioi@mail.doshisha.ac.jp

Supplementary movie 1

Motion of the asymmetric (left) and symmetric (right) gears in a granular bed. The bead diameter is 0.6 mm, and the vibration frequency is 30 Hz. The real time movie.

Supplementary movie 2

Trajectory of colored beads in a granular bed. The bead diameter is 0.6 mm, and the vibration frequency is 30 Hz. The real time movie.

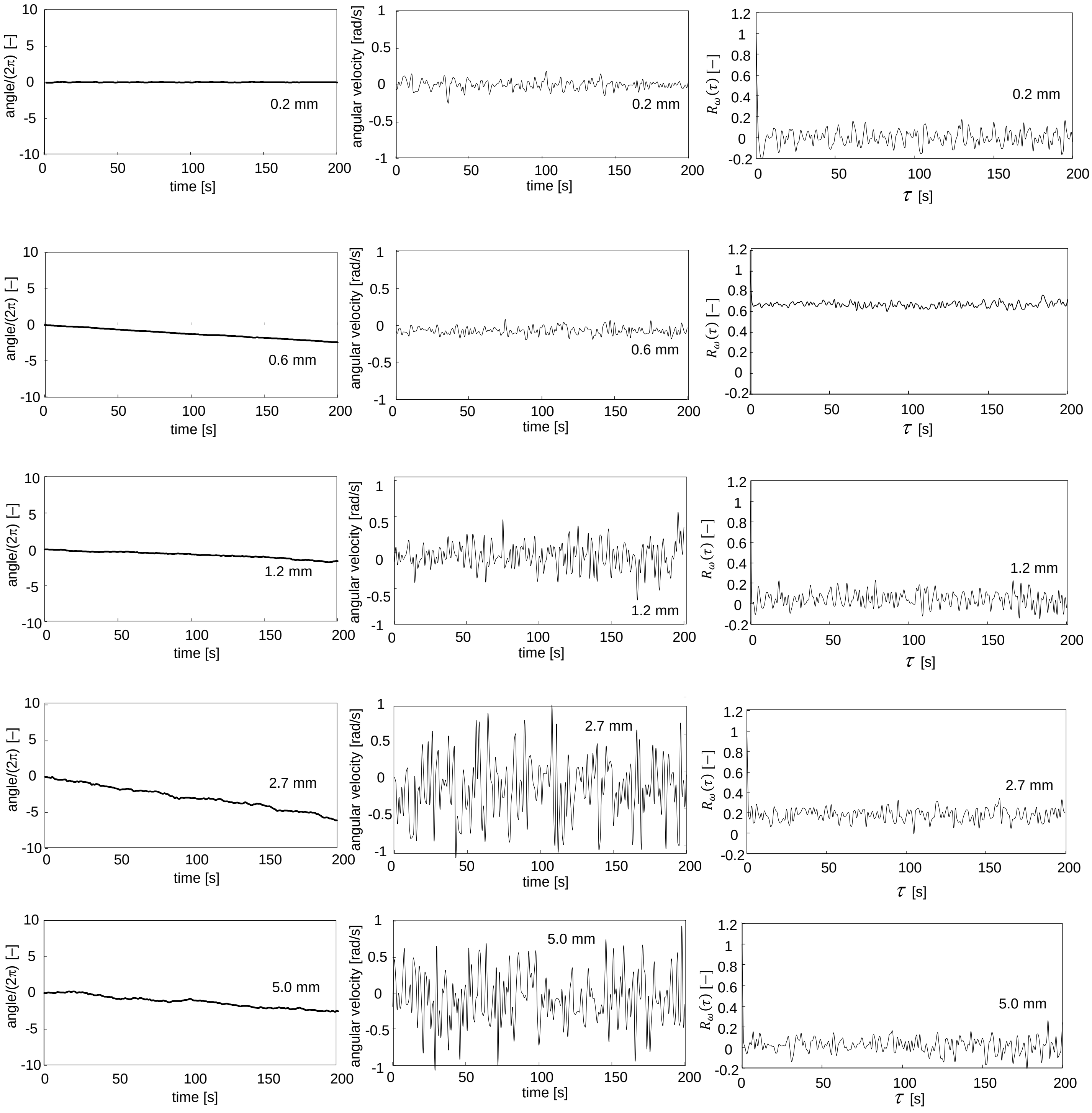

The characteristics of the gear motion with free condition. The azimuth change, the angular velocity and its autocorrelation function for the beads diameter.

Fig.S1

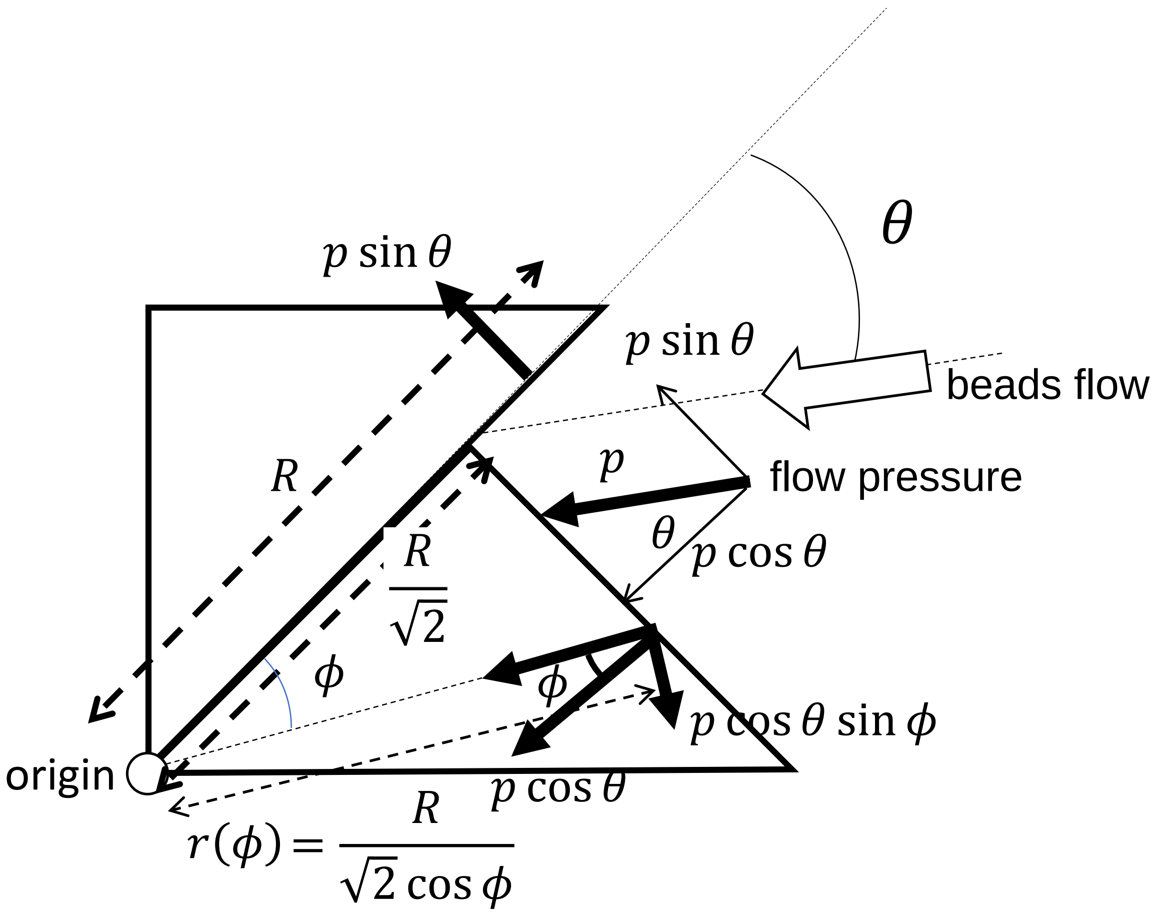

$$\frac{T}{8} = \int_{\frac{R}{\sqrt{2}}}^{R} rp\sin\theta \;\; dr - \int_{0}^{\frac{\pi}{4}} r(\phi)\, p\cos\theta \sin\phi \, r(\phi) \;\; d\phi$$

$$= pR^2\left(\frac{1}{4}\sin\theta - \frac{\sqrt{2}-1}{2}\cos\theta\right)$$

Calculation of the torque for gear spin.

Fig.S2

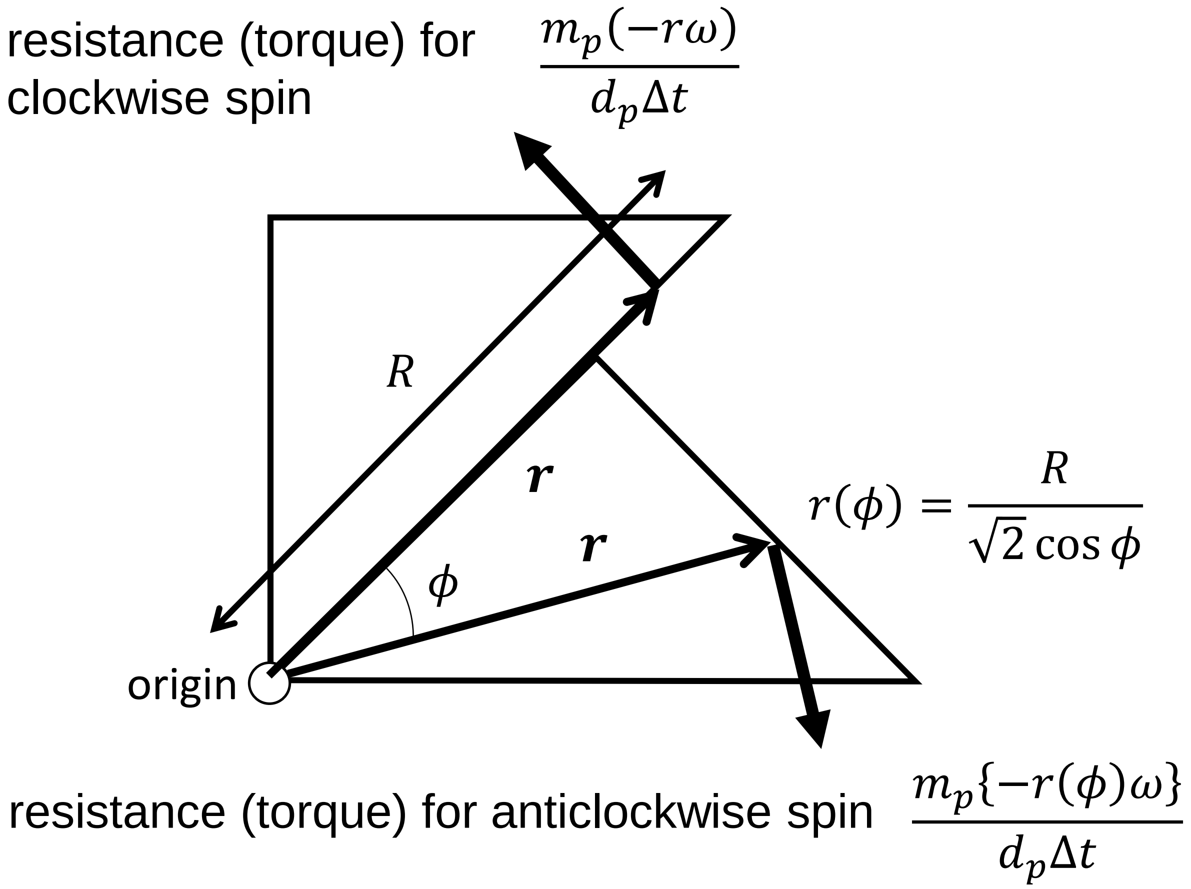

clockwise spin

$$\frac{\text{resistance torque}}{8} = \int_{\frac{R}{\sqrt{2}}}^{R} r\,\frac{m_p(-r\omega)}{d_p\Delta t}\,dr = -\frac{4-\sqrt{2}}{12}\frac{m_pR^3}{d_p\Delta t}\,\omega$$

anticlockwise spin

$$\frac{\text{resistance torque}}{8} = \int_{0}^{\frac{\pi}{4}} r(\phi)\,\frac{m_p\{-r(\phi)\omega\}}{d_p\Delta t}\,r(\phi)\,d\phi$$

$$= -\left\{\frac{1}{4} + \frac{\sqrt{2}}{8}\ln\left(1+\sqrt{2}\right)\right\}\frac{m_pR^3}{d_p\Delta t}\,\omega$$

Calculation of the resistance of spinning gear

Fig.S3

constraint

free

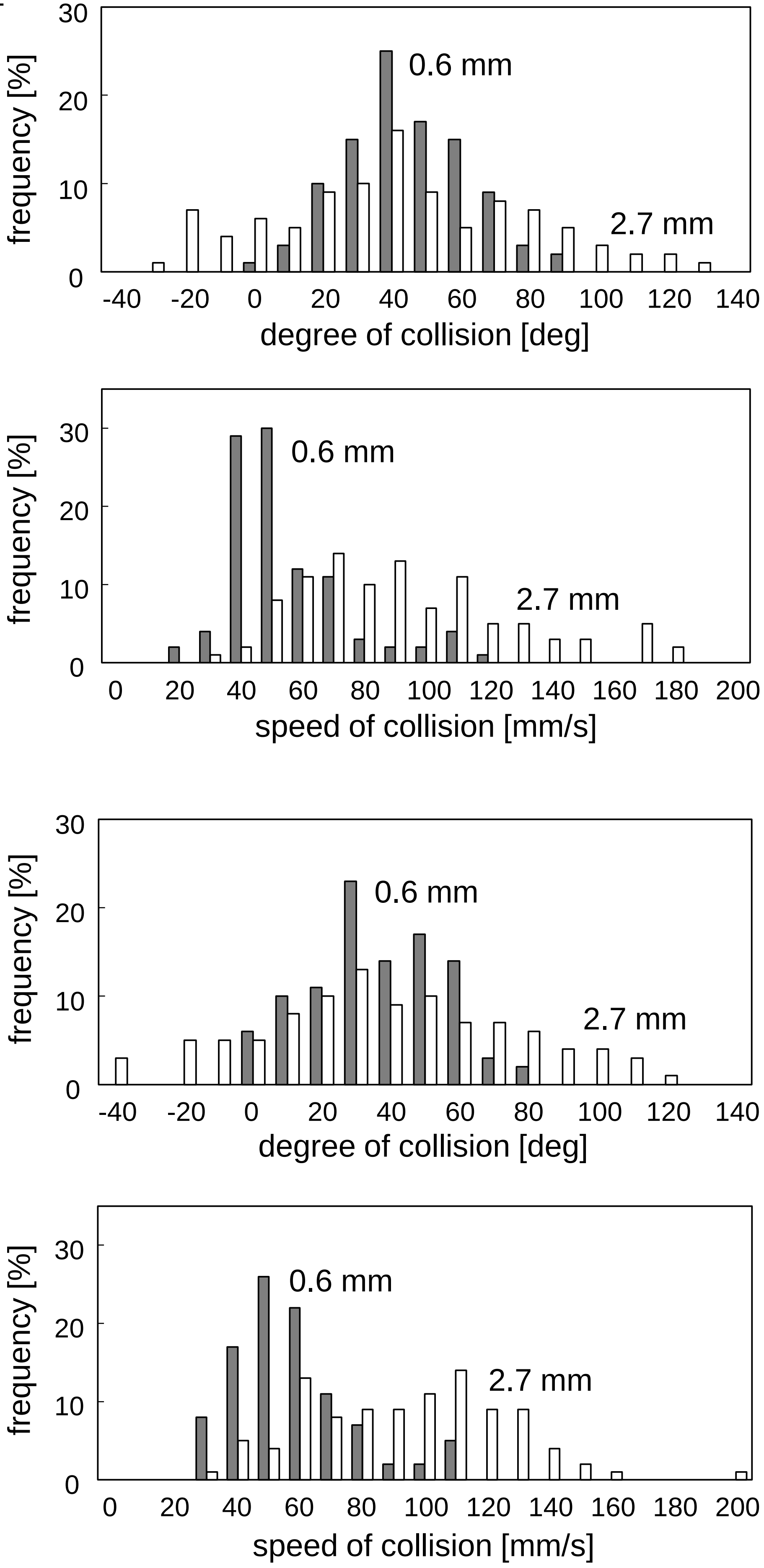

Distribution of collision angle and speed

Fig.S4

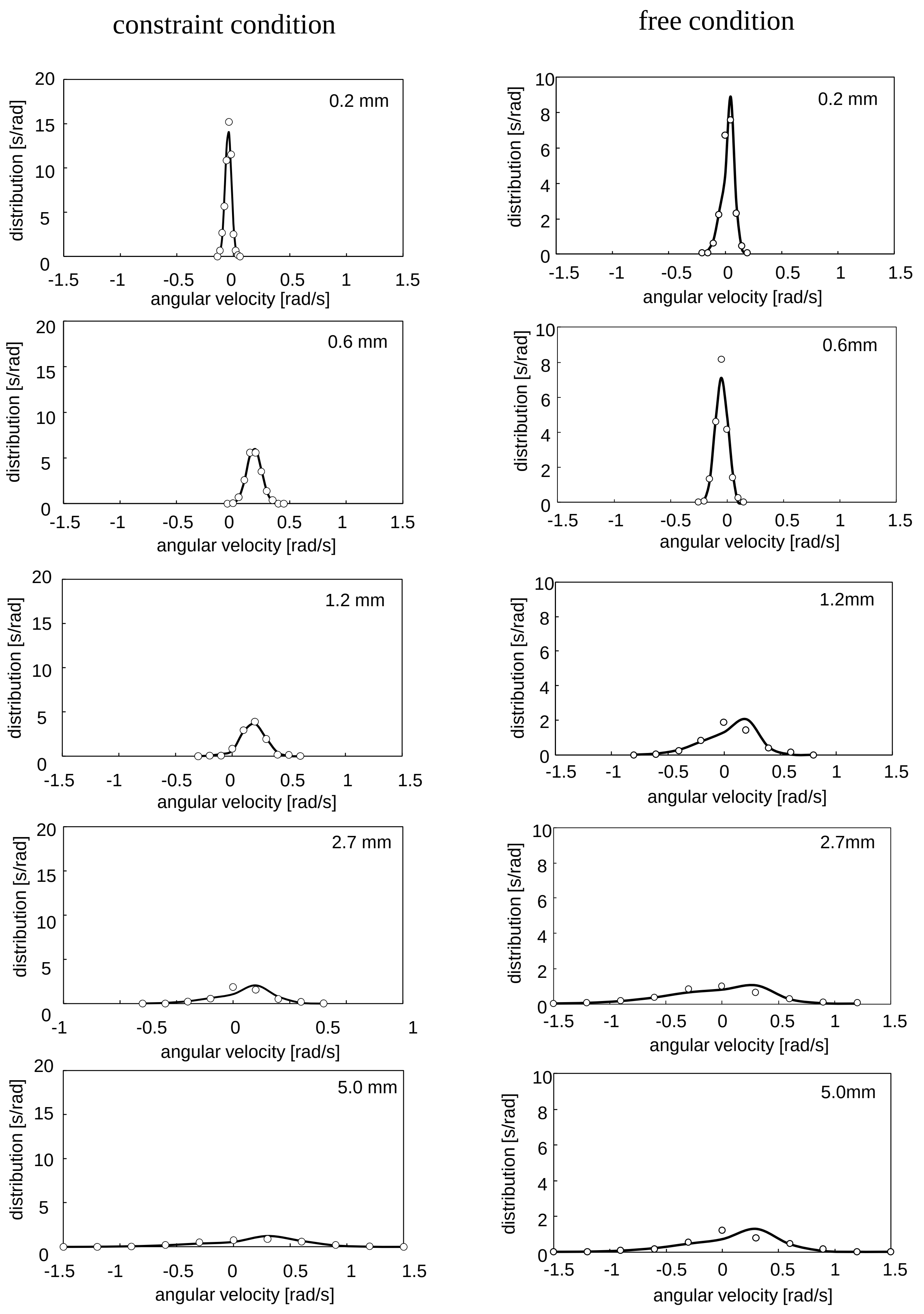

Distribution of angular velocity

Fig. S5

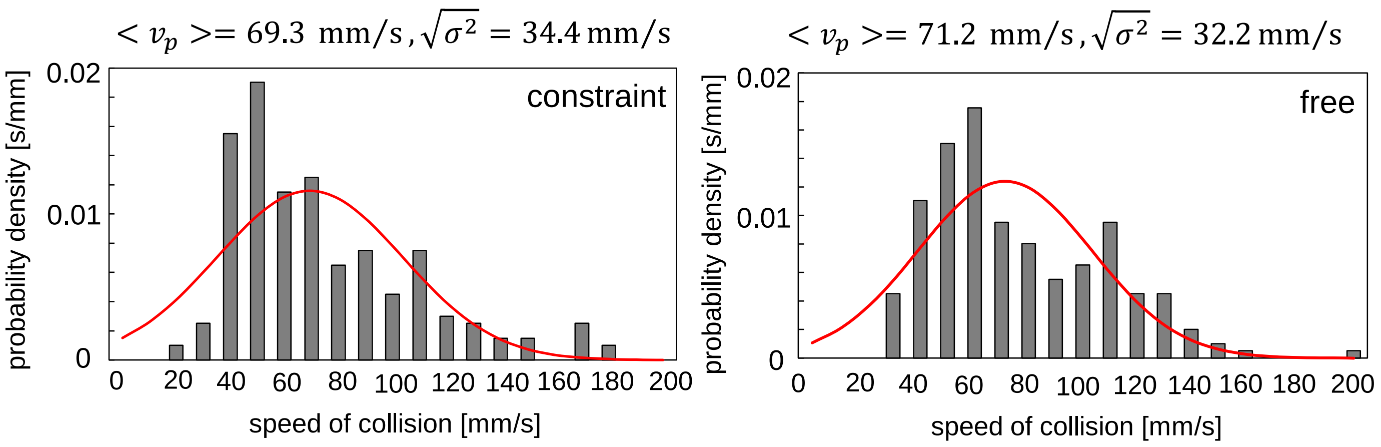

Fitting of the speed distribution by the normal distribution.

Fig. S6
